# Household knowledge, attitudes and practices related to pet contact and associated zoonoses in Ontario, Canada

**DOI:** 10.1186/1471-2458-12-553

**Published:** 2012-07-25

**Authors:** Jason W Stull, Andrew S Peregrine, Jan M Sargeant, J Scott Weese

**Affiliations:** 1 Department of Pathobiology, Ontario Veterinary College, University of Guelph, Guelph, ON, N1G2W1, Canada; 2 Department of Population Medicine, Ontario Veterinary College, University of Guelph, Guelph, ON, N1G2W1, Canada; 3 Centre for Public Health and Zoonoses, University of Guelph, Guelph, ON, N1G2W1, Canada

**Keywords:** Zoonoses, Pet, Education, Knowledge, Bite, Scratch, Canada

## Abstract

**Background:**

Many human infections are transmitted through contact with animals (zoonoses), including household pets. Although pet ownership is common in most countries and non-pet owners may have frequent contact with pets, there is limited knowledge of the public’s pet contact practices and awareness of zoonotic disease risks from pets. The objective of this study was to characterize the general public’s knowledge, attitudes and risks related to pet ownership and animal contact in southern Ontario, Canada.

**Methods:**

A self-administered questionnaire was distributed to individuals at two multi-physician clinics in Waterloo, Ontario, Canada during 2010. A single adult from each household was invited to participate in the study.

**Results:**

Seventy five percent (641/853) of individuals approached completed the questionnaire. Pet ownership and contact were common; 64% of participants had a pet in their household and 37% of non-pet owning households had a member with at least weekly animal contact outside the home. Pet ownership was high (55%) for households with individuals at higher risk for infections (i.e., < 5 yrs, ≥ 65 yrs, immunocompromised). Most respondents (64%) indicated that they had never received information regarding pet-associated disease risks. When given a list of 11 infectious pathogens, respondents were only able to correctly classify just over half on their potential to be transmitted from pets to people (mean 6.4); independently, pet owners and those who recalled receiving information in the past about this topic were able to make significantly more correct identifications. Pet (36%) and non-pet owning households (10%) reported dog or cat bites or scratches during the preceding year. Households with individuals at higher risk for an infection did not differ from the remaining households regarding their perceived disease risk of pets, zoonotic disease knowledge, recall of being asked by their medical provider if they owned any pets, or recall of having received information regarding pet-associated disease risks and preventive measures.

**Conclusions:**

These results suggest that there is a need for accessible zoonotic disease information for both pet and non-owning households, with additional efforts made by veterinary, human and public health personnel. Immediate educational efforts directed toward households with individuals at higher risk to infections are especially needed.

## Background

Pet ownership is common. Although the proportion varies by continent and country, studies indicate that in most countries the majority of households own pets [1-8]. Cats and dogs are the most frequently owned pets, but other species are often reported. A recent study estimated that 56% of Canadian homes have at least one dog or cat, with a minority owning fish (12%), birds (5%), rabbits or hamsters (each 2%), lizards, guinea pigs, snakes, frogs, turtles, ferrets, or gerbils (each 1%) [6]. In addition, people may come into contact with animals outside of their homes such as in public settings (e.g., petting zoos, schools, fairs), through work-related activities, or recreation (e.g., parks), yet little is known about the frequency and nature of this type of animal contact. For the most part, animal ownership and interaction is not discouraged by healthcare professionals, as numerous studies have confirmed the mental and physical benefits of pet ownership and companionship [9], particularly among children, the elderly and immunocompromised individuals [10-16]. Thus, it is not surprising that several studies have indicated that homes with children are more likely to have one or more pets than those without children [3,17-19], and animal ownership practices of immunocompromised individuals appear to be similar to those of the general public [15,20,21]. Despite these benefits, there are also potential health hazards associated with pet ownership and contact.

Animal bites are a serious public health problem, with an estimated 316,000 U.S. emergency room visits for a dog bite-related injury in 2008 (rate: 104 visits per 100,000 population) [22]. Dogs are responsible for approximately 80% of all bites, cats accounting for less than 20% and other pet species and wildlife responsible for the remainder [23]. Bites may lead to painful disfiguring wounds, infection, altered function of the affected area, and rarely death. Animal scratches can also have important physical consequences depending on the area of the body affected. In addition to the physical damage of bites and scratches, pets can transmit pathogens to people (zoonoses). People can acquire pet-associated zoonotic organisms through the skin and mucous membranes (via animal bites, scratches, or other direct contact), contact with animal saliva, urine and other body fluids or secretions, ingestion of animal fecal material, inhalation of infectious aerosols or droplets, and through arthropods and other invertebrate vectors [24]. Although any exposed person can become infected with a zoonotic pathogen, risks are particularly high for those with a compromised or incompletely developed immune system, such as the young (< 5 yrs), elderly (≥ 65 yrs), pregnant and those with immune function-reducing conditions or treatments, including diabetes, cancer, infection with human immunodeficiency virus (HIV), splenectomy and radiation therapy [24,25]. The increased disease risk for children is additionally imparted through closer physical contact with household animals, reduced hand hygiene and behaviours that include pica and exploration of the environment through mouthing. Not only are these groups at increased risk for infection with a zoonotic pathogen, infection with many zoonotic pathogens is more likely to result in severe disease in high risk groups [24,25]. Pets often have frequent, close interactions with household members, such as licking of hands and sleeping in beds [26], which can further increase pet-associated disease risks.

Many of the disease risks that occur with pet contact can be eliminated or reduced through simple measures, such as hand hygiene, proper animal husbandry and altered animal-contact behaviours. In order for infection prevention to be successful, however, individuals in contact with animals must be aware of these disease risks. Thus, awareness of zoonotic disease risk is a prerequisite for effective disease prevention. To-date, few studies have evaluated the general public’s knowledge of pet-associated zoonoses [27,28]. A study conducted in 1986 revealed that many individuals in the general population lacked even basic knowledge about zoonotic diseases (e.g., only 63% of household heads in De Kalb County, Georgia, USA believed pathogens from pets could be transmitted to humans) [28]. A more recent study (2008) supported the earlier study’s findings of poor zoonotic disease knowledge by the general public (e.g., only 56% of dog owners in Brazos County, Texas, USA knew intestinal helminths could be transmitted from dogs to humans) [27]. Both studies were limited in their geographic scope and did not integrate human disease risk status (i.e., extremes of age, immunocompromised), animal contact-related attitudes, and zoonotic disease knowledge and risks. In addition, most previous studies have only measured animal contact through pet ownership and did not include animal contact outside of the household. The objective of this study was to further characterize the general public’s household knowledge, attitudes and risks related to pet ownership and animal contact. Furthermore, this study aimed to determine if those households with individuals at increased risk of disease differ in knowledge, attitudes, or practices from the remainder of the public.

## Methods

### Study location and selection of study sites

The site chosen for this study was the region of Waterloo, located in southern Ontario, Canada. The Waterloo Region is composed of three urban and four rural municipalities. This region was selected as it includes both rural and urban settings and its population has similar demographics to the Province of Ontario and Canada as a whole. As of 2006, this region had a population of approximately 478,000 people in 178,000 private households. The median age was 36.4 yrs, with 81% of the population aged 15 yrs and over, 6% less than 5 yrs, 12% greater than 64 yrs, 22% immigrants and 13% of a visible minority (non-Caucasian in race or non-white in colour). Average household size was 2.6 persons, with a median annual income of $65,000 CAD [29]. These statistics are similar to those for the Province of Ontario and all of Canada, with the exception of a lower prevalence of visible minorities (23% for Ontario, 16% for Canada) and a higher household median annual income ($60,000 for Ontario, $54,000 for Canada) [29].

In Canada, all residents receive medically necessary healthcare services at no charge [30], thereby reducing the potential of biasing a study population drawn from healthcare facilities. A convenience sample of multi-doctor general practice physician offices located within the Waterloo region were approached to participate in the study. Two practices, each with at least 4 physicians, agreed to participate and were selected (clinic A in Kitchener, Ontario and clinic B in Cambridge, Ontario).

### Data collection

During 4 weeks in October and November, 2010, individuals present in the waiting areas of the participating clinics between 9 am and 4 pm were individually approached by one of the authors (JWS) and invited to take part in the study. Individuals were eligible to participate if they were at least 18 yrs of age, able to read and speak English and no one from their household had previously taken part in the study. Individuals who appeared to be in distress or pain were not approached. A single member from each household was asked to complete an anonymous, confidential 10-minute self-administered written questionnaire on-site or at a later time. Individuals in the latter group were provided a stamped, addressed envelope to return the questionnaire.

A 5-page questionnaire was developed with guidance from epidemiologists, veterinarians, physicians and zoonotic disease experts. The questionnaire was piloted on six members of the public with varying zoonotic disease backgrounds and revised accordingly. The final questionnaire utilized closed-ended, primarily multiple choice, questions (Additional File 1: Doc1). It gathered both individual and household-level data including demographics, pet and animal contact-related attitudes, respondent knowledge of zoonotic diseases and sources for such information, the occurrence of animal contact and pet-associated zoonotic disease and injury, health status of household members (i.e., ever diagnosed with HIV/AIDS, diabetes, cancer, hepatic cirrhosis, or other cause for immune dysfunction) and types of pets (if any) in the household. The immigrant generation status of the respondent, a designation indicating if they or their parents were born outside Canada, was assigned based on established criteria [31]. Demographic, health and injury information was requested for each individual who lived in the household at the time of completing the questionnaire. The respondents’ general knowledge of zoonotic diseases was assessed by providing a list of infectious pathogens, diseases and syndromes (rabies, intestinal worms, human immunodeficiency virus (HIV)/acquired immune deficiency syndrome (AIDS), distemper, *SalmonellaGiardia*, hepatitis, infectious diarrhea, ringworm, methicillin-resistant *Staphylococcus aureus* (MRSA), measles) and asking the respondents to indicate which they believed could be transmitted from pets to people. Components of this list were chosen based on their use in previous studies [27,28] as well as the intent to capture zoonotic and non-zoonotic pathogens that are encountered or should be discussed with clients/patients with some frequency in veterinary or human medicine. The syndrome infectious diarrhea was included as it was used in a previous study [28] and to assess the ability of respondents to recognize an important syndrome verses a specific pathogen. A zoonotic disease knowledge score was calculated by summing the frequency a participant correctly classified the diseases as pet-associated zoonoses (min-max: 0–11). A non-pet household survey was created by discarding questions specific to current pet ownership (Additional File 2: Doc2). After agreeing to participate in the study, individuals were asked if they currently had any pets in their household and then provided with the appropriate questionnaire. Household pets included pets that were indoor only, outdoor only, and those that spent time both indoor and outdoor. The study was approved by the University of Guelph’s Research Ethics Board.

### Data analysis

Data were entered into an Access database (Microsoft Corp., Redmond, WA, USA) and exported and analyzed using Intercooled Stata version 10.1 (Stata Corp., College Station, TX, USA). Descriptive statistics were computed for all variables and stratified by pet ownership. Blank and “don’t know” responses were excluded from analyses. Statistical associations were assessed between pet ownership and each variable. Associations with categorical variables were assessed using Pearson *χ*^2^ test or, if any expected cell value was ≤ 1 or 20% or more expected cell values were ≤ 5, Fisher’s exact test [32]. The non-parametric Cuzick test of trend was used for assessing associations with ordinal variables. Student’s *t*-test assuming unequal variances or, if a non-normal distribution, the Mann–Whitney rank sum test was used for assessing associations with continuous variables. The associations between respondents’ zoonotic disease knowledge score and the categorical variables (1) level of comfort with their zoonotic disease knowledge, and (2) previous receipt of information about pet-associated zoonoses, were assessed using the one-way analysis of variance (ANOVA) test and Student’s *t*-test assuming unequal variances (or Mann–Whitney rank sum test if non-normal distribution), respectively. Multiple comparisons with Bonferroni adjustment were performed following a statically significant ANOVA test. The associations between the presence of individuals at higher risk to infections (i.e., < 5 yrs, ≥ 65 yrs, immunocompromised) in the household and select variables were assessed as stated above for continuous and categorical variables, while stratifying on pet ownership as a potential confounder. When applicable, 95% confidence intervals (CI) were calculated using binomial exact methods. Statistical significance was based on a *P*-value <0.05.

## Results

During the 4-week period in which the survey was administered, 641 of the 853 individuals who were approached (75.1%) completed the survey (114 refused, 98 did not return or complete the survey). The proportion responding was similar for both clinics [clinic A – 374/490 (76.3%), clinic B – 267/363 (73.6%)]. Twenty individuals were not approached as they appeared to be in pain or distress. Four hundred and eight respondents (63.7%; 95% CI: 59.8, 67.4) reported having one or more household pets. Dogs were the most frequently reported owned species (42.8% of respondents; 95% CI: 38.9, 46.7), followed by cats (29.8%; 95% CI: 26.3, 33.5), fish (8.3%; 95% CI: 6.3, 10.7), exotic companion animals, such as gerbils and rabbits (4.5%; 95% CI: 3.1, 6.4), reptiles and birds (each 3.4%; 95% CI: 2.2, 5.2). Cats or dogs were owned by 93% (379/408) of the pet owners.

### Demographics

Demographics on a total of 1,971 household members were provided by the respondents. Demographics varied slightly from reported statistics for the region, with a median age of 33 yrs, 77% aged 15 yrs and older, 8% less than 5 yrs, 8% greater than 64 yrs, 6% non-white, mean household size of 3.1 persons and median category for annual household income between $80,000 and $120,000 CAD. Statistically significant associations were observed between pet ownership and respondent sex, age, race, residence classification, number of individuals living in the household and presence of individuals at increased risk for acquiring an infectious disease living in the household, including elderly and those with an immunocompromising condition (Table 1). Pet owning households tended to have more individuals, live in suburban or rural areas and were less likely to have higher risk individuals living in the household (including fewer elderly and individuals with an immunocompromising condition) as compared to non-pet owning households. Although households with elderly and immunocompromised individuals were less likely to have pets than households without these higher risk individuals, pet ownership remained common (47% and 55%, respectively). Diabetes and cancer (53% and 27% of immunocompromised respondents, respectively) were the most frequently reported immunocompromising conditions, followed by “other cause” (9%); more than one cause (most frequently diabetes and cancer) were listed by the remaining immunocompromised respondents (11%). Respondents from pet owning households were statistically more likely to be female, younger and Caucasian as compared to non-pet owning households.

**Table 1:** Demographics for survey respondents (Ontario, Canada; N = 641)

**Variables**	**Number respondents (N)**	**Pet currently present in household**		***P*-value**^**1**^
		**Yes, N (%)**	**No, N (%)**	
Respondent’s sex	638			0.011
Male		93 (56)	74 (44)	
Female		314 (67)	157 (33)	
Respondent’s age (years)	637			< 0.001^2^
Mean (SD)		44.1 (14.1)	48.8 (17.2)	
Median (range)		44 (17–89)	49 (16–86)	
Respondent’s race	616			< 0.001
White		380 (66)	200 (34)	
Other^3^		11 (31)	25 (69)	
Immigrant generation status of respondent^4^	595			0.27
First		45 (58)	32 (42)	
Second		61 (70)	26 (30)	
Third or more		269 (62)	162 (38)	
Self-reported residence classification	632			< 0.001
City		263 (62)	161 (38)	
Suburban		72 (75)	24 (25)	
Town		31 (48)	34 (52)	
Rural		38 (81)	9 (19)	
Total household income during past 12 months (before taxes and deductions)				
	532			0.06^5^
Less than Can$20,000		14 (61)	9 (39)	
Between Can$20,000 and Can$39,999		46 (63)	27 (37)	
Between Can$40,000 and Can$79,999		96 (58)	70 (42)	
Between Can$80,000 and Can$120,000		104 (68)	49 (32)	
More than Can$120,000		83 (71)	34 (29)	
Highest level of education attained by anyone currently living in household				
	607			0.34^6^
Elementary school		1 (25)	3 (75)	
High school certificate, diploma, or equivalent		66 (68)	31 (32)	
College, trade or other non-university certificate or diploma		141 (64)	79 (36)	
University certificate, diploma or degree		180 (63)	106 (37)	
Number of individuals living in household	641			<0.001^2^
Mean (SD)		3.2 (1.4)	2.8 (1.5)	
Median (range)		3 (1–9)	2 (1–8)	
Children under 16 yrs living in household	641			0.10
Yes		183 (67)	89 (33)	
No		225 (61)	144 (39)	
Children under 5 yrs living in household	641			0.24
Yes		64 (59)	45 (41)	
No		344 (65)	188 (35)	
Adults ≥ 65 yrs living in household	641			<0.001
Yes		56 (47)	63 (53)	
No		352 (67)	170 (33)	
Anyone currently living in household ever diagnosed with an immunocompromising condition				
	540			0.008
Yes		94 (55)	78 (45)	
No		245 (67)	123 (33)	
One or more individuals living in household < 5 yrs, ≥ 65 yrs, or ever diagnosed with an immunocompromising condition				
	572			<0.001
Yes		184 (55)	151 (45)	
No		173 (73)	64 (27)	

^1^*P*-value for Pearson *χ*^2^ test, unless otherwise stated.

^2^ Mann–Whitney rank sum test.

^3^ Includes Arab, Black, Chinese, Filipino, Japanese, Latin American, South Asian, Southeast Asian, Western Asian, Other.

^4^ First generation, defined as born outside Canada; second generation, defined as born inside Canada with at least one parent born outside Canada; third generation or more, defined as born inside Canada with both parents born inside Canada [31].

^5^ Non-parametric Cuzick test of trend.

^6^ Fisher’s exact test.

### Pet and animal contact-related attitudes

Perceptions of pet ownership and zoonotic disease risk varied significantly between pet and non-pet owning households (Table 2). Amongst respondents from pet owning households, significantly less concern was reported for pet-associated disease for themselves and household children than in non-pet owning households. For both groups, a high percentage of respondents (≥ 30%) reported they were not at all concerned about pet-associated disease. Households with young children (< 5 yrs) and households with older children (5–15 yrs) did not differ significantly in their concern for household children acquiring pathogens from pets (Fisher’s exact test: pet owner: *P* = 0.13, non-pet owner: *P* = 0.32). Approximately one-third of respondents (n = 214) listed one or more pet-associated diseases that they considered to be of greatest concern to them. The majority of respondents were most concerned about rabies (n = 84; 39%) or endo/ecto parasite-derived disease (n = 85; 40%). Bacterial (n = 26; 12%) and viral (excluding rabies) pathogens (n = 17; 8%) were less frequently listed, with *Salmonella*, *Escherichia coli* and influenza virus being the most common in those categories. Allergies/asthma (n = 11; 5%) and ringworm (n = 7; 3%) were also listed.

**Table 2:** Pet and animal contact-related attitudes of survey respondents (Ontario, Canada; N = 641)

**Variables**	**Number respondents (N)**	**Pet currently present in household**		***P*-value**^**2**^
		**Yes, N (%)**^**1**^	**No, N (%)**^**1**^	
How concerned are you that household children could catch a disease from your pets or pets of friends or family?^3^				
	268			0.001
Very concerned		9 (5)	10 (12)	
Concerned		9 (5)	8 (9)	
Somewhat concerned		14 (8)	13 (15)	
Minimally concerned		69 (38)	29 (34)	
Not at all concerned		81 (45)	26 (30)	
How concerned are you that you could catch a disease from your pets or pets of friends or family?^3^				
	612			<0.001
Very concerned		12 (3)	14 (7)	
Concerned		18 (4)	21 (10)	
Somewhat concerned		29 (7)	29 (14)	
Minimally concerned		140 (35)	59 (28)	
Not at all concerned		204 (51)	86 (41)	
Pets are an important part of the family				
	618			< 0.001
Strongly agree		340 (84)	76 (36)	
Somewhat agree		62 (15)	101 (48)	
Somewhat disagree		4 (1)	20 (9)	
Strongly disagree		1 (0.3)	14 (7)	
Benefits of owning a pet are greater than any health risks that occur with owning a pet				
	601			<0.001
Strongly agree		242 (61)	56 (28)	
Somewhat agree		108 (27)	79 (39)	
Somewhat disagree		37 (9)	37 (18)	
Strongly disagree		11 (3)	31 (15)	
Removal of one or more of my pets would negatively affect people in my household				
	401			
Strongly agree		300 (75)	----	
Somewhat agree		73 (18)	----	
Somewhat disagree		18 (4)	----	
Strongly disagree		10 (2)	----	

^1^ Percentages in column may not sum to 100 % due to rounding.

^2^*P*-value for non-parametric Cuzick test of trend.

^3^ Category “no contact with pets” removed from analysis due to low frequency.

Households with pets were significantly more likely to consider pets an important part of a family and believed the benefits of owning a pet were greater than pet-associated health risks (Table 2). Although less frequent, the majority of non-pet owning households stated they agreed or strongly agreed that pets are an important part of the family (84%) and that the benefits of pet ownership outweigh any health risks (67%). Households with high risk individuals (i.e., <5 yrs, ≥ 65 yrs, immunocompromised) and without these individuals did not differ significantly in their beliefs regarding the benefits and risks of pet ownership (Pearson *χ*^2^: pet owner: *P* = 0.54, non-pet owner: *P* = 0.61). Ninety-three percent of pet-owners stated that removal of one or more pets would negatively affect people in their household.

### Zoonotic disease knowledge and educational sources

When provided a list of 11 infectious pathogens, diseases and syndromes, participants were able to correctly classify just over half on their potential to be transmitted from pets to people [mean zoonotic disease knowledge score (SD) = 6.4 (1.4)], with a significant difference between pet and non-pet owners (6.5, 6.2 respectively; Table 3). Households with and without high risk individuals did not differ significantly in their mean zoonotic disease knowledge score (*t*-test: pet owner: *P* = 0.15, non-pet owner: *P* = 0.64). Rabies and measles were most often classified correctly (≥ 95%), while *Giardia* and MRSA were least frequently classified correctly (≤ 9%). Significant differences in correct classification between pet and non-pet owners were observed for several of the pathogens, diseases and syndromes, including *Salmonella*, *Giardia* and hepatitis, with pet owners more frequently correctly categorising *Salmonella* and *Giardia*.

**Table 3:** Zoonotic disease knowledge and educational sources of respondents (Ontario, Canada; N = 641)

**Variables**	**Number respondents (N)**	**Pet currently present in household**		***P*-value**^**2**^
		**Yes, N (%)**^**1**^	**No, N (%)**^**1**^	
Which of the following diseases do you think can be transmitted from pets to people?				
	599			
Rabies*		374 (96)	198 (94)	0.15
Intestinal worms*		214 (55)	103 (49)	0.14
HIV/AIDS		43 (11)	14 (7)	0.076
Distemper		43 (11)	19 (9)	0.43
Salmonella*		146 (38)	51 (24)	0.001
Giardia*		37 (10)	9 (4)	0.021
Hepatitis		52 (13)	17 (8)	0.050
Infectious diarrhea*		98 (25)	47 (22)	0.42
Ringworm*		215 (55)	103 (49)	0.12
Methicillin-resistant *Staphylococcus aureus* (MRSA)*		36 (9)	18 (9)	0.76
Measles		14 (4)	12 (6)	0.23
Mean knowledge score (SD)^3^		6.5 (1.4)	6.2 (1.4)	0.023^4^
I am comfortable with my level of understanding of possible diseases that can occur with pet contact				
	577			<0.001^5^
Strongly agree		136 (35)	32 (17)	
Somewhat agree		146 (38)	90 (47)	
Somewhat disagree		65 (17)	43 (22)	
Strongly disagree		37 (10)	28 (15)	
I am comfortable with my level of understanding of ways to reduce diseases that can occur with pet contact				
	567			<0.001^5^
Strongly agree		121 (32)	26 (14)	
Somewhat agree		143 (38)	81 (42)	
Somewhat disagree		72 (19)	48 (25)	
Strongly disagree		40 (11)	36 (19)	
Medical doctors or their staff ever discussed the possible benefits of owning or keeping a pet				
	630			0.003
Yes		31 (8)	7 (3)	
No		338 (84)	213 (93)	
Don’t remember		33 (8)	8 (4)	
Medical doctors or their staff ever asked if you owned any pets				
	630			0.029
Yes		99 (25)	39 (17)	
No		243 (60)	139 (61)	
Don’t remember		61 (15)	49 (22)	
Ever received information from any source about diseases that you can get from pets or precautions to take with pets to reduce the risk of disease				
	630			< 0.001
Yes		145 (36)	48 (21)	
No		227 (56)	162 (72)	
Don’t remember		33 (8)	15 (7)	
Sources that provided this information^6^	190			NP
Family physician		28 (20)	14 (29)	
Specialist physician		13 (9)	2 (4)	
Nursing staff		6 (4)	4 (8)	
Public health personnel		13 (9)	9 (19)	
Veterinarian		103 (73)	14 (29)	
Pet store		27 (19)	6 (13)	
Animal breeder		14 (10)	1 (2)	
Friends/relative		27 (19)	17 (35)	
Internet		45 (32)	18 (38)	
Books		41 (29)	12 (25)	
Television/ newspaper/ magazines		29 (20)	12 (25)	
Other		5 (4)	3 (6)	
Most useful source (when 2 or more)	100			NP
Family physician		1 (1)	2 (8)	
Specialist physician		4 (5)	1 (4)	
Public health personnel		1 (1)	1 (4)	
Veterinarian		45 (59)	7 (29)	
Pet store		1 (1)	0	
Animal breeder		3 (4)	0	
Friends/relative		2 (3)	4 (17)	
Internet		8 (11)	5 (21)	
Books		7 (9)	2 (8)	
Television/ newspaper/ magazines		3 (4)	1 (4)	
Other		1 (1)	1 (4)	
Who do you believe should be responsible for providing information about diseases that can occur with pet contact?^6^				
	627			NP
Family physician		190 (47)	121 (55)	
Specialist physician		26 (6)	15 (7)	
Nursing staff		28 (7)	20 (9)	
Public health personnel		127 (31)	120 (54)	
Veterinarian		350 (86)	125 (56)	
Breeder/ pet store/ shelter		18 (4)	10 (5)	
Self		15 (4)	0	
Media		3 (1)	8 (4)	
None		34 (8)	22 (10)	
Other		2 (0)	2 (1)	

^1^ Percentages in column may not sum to 100 % due to rounding.

^2^*P*-value for Pearson *χ*^2^ test, unless otherwise stated.

^3^ Calculated as the frequency participants correctly classified the listed diseases as transmitted from pets to people (Min-Max possible score: 0–11).

^4^ Student’s *t*-test, assuming unequal variances.

^5^ Non-parametric Cuzick test of trend.

^6^ Categories sum to greater than 100 % as some participants listed more than one category.

* Pathogens/syndromes transmitted from pets to people.

NP: Statistical analysis not performed.

Seventy percent of respondents were comfortable with their level of understanding of zoonotic diseases acquired through pet contact, while 65% were comfortable with their level of understanding of ways to prevent such diseases (Table 3). Significant associations were observed with pet ownership for both disease knowledge and prevention comfort levels, with pet owners being more likely to be comfortable with their level of understanding. Households with and without high risk individuals did not differ significantly in their disease knowledge and prevention comfort levels (Pearson *χ*^2^: pet owner: *P* = 0.20, *P* = 0.67; non-pet owner: *P* = 0.24, *P* = 0.26, respectively). For pet owners, level of comfort with understanding of zoonotic disease was significantly positively associated with zoonotic disease knowledge score (ANOVA; *P* = 0.034); those who were more comfortable with their knowledge-base had higher knowledge scores than those who were less comfortable. However, in pair-wise comparisons, only the two extreme comfort levels, “strongly agree” (mean score = 6.7) and “strongly disagree” (5.9), differed significantly (*P* = 0.020). These associations were not observed for non-pet owners (*P* = 0.47).

A minority of respondents (22%) reported ever being asked by a medical doctor or their staff if they owned a pet, while 6% reported a medical doctor or staff had at some point discussed the possible benefits of pet ownership; for both, pet owners more frequently reported these findings than non-pet owners (Table 3). Households with and without high risk individuals did not differ significantly in their reporting of ever being asked if they owned a pet (Pearson *χ*^2^: pet owner: *P* = 0.33; non-pet owner: *P* = 0.80). Approximately 30% of respondents reported ever receiving information from any source about diseases acquired from pets or precautions to take with pets to reduce the risk of these diseases, with a significantly higher percentage among pet owners as compared to non-pet owners (36%, 21%). Households with and without high risk individuals did not differ significantly in reporting they had ever received this information (Pearson *χ*^2^: pet owner: *P* = 0.89; non-pet owner: *P* = 0.96). Respondents who reported they had received information in the past had a significantly higher mean zoonotic disease knowledge score than those who reported they had not [*t*-test; pet owners: 6.7 vs. 6.3 (*P* = 0.018); non-pet owners: 6.8 vs. 6.1 (*P* = 0.008)]. As the content of pet-associated disease education typically focuses only on diseases that are zoonotic, without discussing diseases that are not, analysis was repeated calculating the zoonotic disease knowledge score for only the subset (7) of the pathogens, diseases and syndromes that are zoonotic. A similar finding was observed, with those who reported receiving information having a significantly higher rank sum knowledge score than those who reported they had not [Mann–Whitney rank sum test; pet owners’ median scores: 3 vs. 3 (*P* = 0.002); non-pet owners’ median scores: 3 vs. 2 (*P* = 0.0006)]. Sources providing this information varied between pet and non-pet owners, with veterinarians (73%), the Internet (32%), books (29%), television/newspaper/magazine (20%) and family physicians (20%) being the most common for pet owners; the Internet (38%), friends/relatives (35%), family physicians and veterinarians (each 29%) and books and television/newspaper/magazine (each 25%) most commonly reported for non-pet owners. When two or more sources were listed, pet and non-pet owners named veterinarians and the Internet as the most “useful” sources. Of the 82% of pet-owners who had taken a pet to the veterinarian in the past 12 months, only 27% reported having ever received information from a veterinarian about pet-associated zoonotic diseases. Pet and non-pet owners believed veterinarians, family physicians and public health personnel (in that order) should be responsible for providing zoonotic disease information to the public, with 86% of pet-owners and 56% of non-pet owners looking toward veterinarians for this information.

### Zoonotic disease risks

Four percent of respondents reported that someone in their household had at some point acquired a disease from a pet, with no statistical difference between pet and non-pet owners (Table 4). The most frequently reported pathogen was ringworm (40%), followed by “worms” (12%). During the preceding 12 months, 36% of pet-owners and 10% of non-pet owners (*P* < 0.001) claimed someone in their household had been bitten or scratched by a dog or cat, resulting in a wound where the skin was broken. Scratches were more common than bites, with the household pet often involved in pet owning households. Thirty one individuals from 27 households were reported being bitten by a dog during the preceding 12-months (number per household: median = 1; range = 1-2), corresponding to 1.6% (31/1971) of all reported household members.

**Table 4:** Zoonotic disease risks for respondent households (Ontario, Canada; N = 641)

**Variables**	**Number respondents (N)**	**Pet currently present in household**		***P*-value**^**1**^
		**Yes, N (%)**	**No, N (%)**	
Has anyone in your household ever caught a disease from a pet?				
	633			0.74
Yes		14 (3)	9 (4)	
No		392 (97)	218 (96)	
During the past 12 months, has anyone in your household been bitten or scratched by any dog or cat, where the skin was broken?				
	616			<0.001
Yes		144 (36)	22 (10)	
No		252 (64)	198 (90)	
Type of injury^2^	166			NP
Scratched by own dog		53 (37)	3 (14)	
Scratched by another dog		14 (10)	7 (32)	
Bitten by own dog		15 (10)	1 (5)	
Bitten by another dog		5 (3)	7 (32)	
Scratched by own cat		68 (47)	3 (14)	
Scratched by another cat		14 (10)	8 (36)	
Bitten by own cat		24 (17)	2 (9)	
Bitten by another cat		6 (4)	2 (9)	
Does anyone in your household regularly (at least weekly) have physical contact with animals in places outside of the home?				
	573			0.68
Yes		128 (35)	76 (37)	
No		238 (65)	131 (63)	
Type of animal/location (subset listed)	204			NP
Dog and/or cat at friend’s or relative’s residence		80 (63)	51 (67)	
Farm animals at work, lessons, or friend’s residence		17 (13)	2 (3)	
Reptile at friend’s residence or work		3 (2)	1 (1)	
Wildlife		1 (1)	1 (1)	
Plan on acquiring a new pet in the next year				
	621			0.95
Yes		41 (10)	24 (11)	
No		353 (90)	203 (89)	

^1^*P*-value for Pearson *χ*^2^ test.

^2^ Categories sum to greater than 100 % as some participants listed more than one category.

NP: Statistical analysis not performed.

Thirty six percent of all respondents stated that someone in their household had physical contact with animals outside the home on a weekly basis. Pet ownership was not associated with animal contact outside the house. The most frequently reported type of contact outside of the home involved cats or dogs at a friend’s or relative’s residence (63% pet owners; 67% non-pet owners), followed by farm animals (13% pet owners; 3% non-pet owners). Contact with reptiles or wildlife (e.g., raccoons, skunks) outside of the home were infrequently reported for both pet and non-pet owners (≤ 2% each). Approximately 10% of both pet and non-pet owners planned on acquiring a new pet in the next year.

## Discussion

This study aimed to characterize household knowledge, attitudes and risks related to pet ownership and animal contact in Ontario, Canada. Despite the importance of this topic due to the potential injury and disease risks posed by pets, the high proportion of households that own pets, and the close interaction pets often have with household members, few studies [27,28] have evaluated this area. Furthermore, this study is unique in that it broadly addresses animal contact within and outside the home, and integrates household demographics, including human disease risk status (i.e., extremes of age, immunocompromised), animal contact-related attitudes and zoonotic disease knowledge and risks.

The animal ownership patterns we observed were consistent with those previously reported for the surrounding area [19] and country [6], with over 63% of households having one or more pets. Similar to a previous study [33], over 75% of respondent households had one or more members that had frequent animal contact through household pets, animals outside the home, or both. These results highlight the common occurrence of direct animal exposure for the public. The perceived benefits from pet ownership and contact were evident as both pet and non-pet owners believed that the benefits of pet ownership outweigh disease risks.

In order to assess zoonotic disease knowledge, respondents were provided a list of infectious pathogens, diseases and syndromes and asked to indicate which were transmitted from pets to people. The decision as to which of the listed pathogens, diseases and syndromes were pet-associated was based on the best available knowledge at the time the survey was administered. Pathogens that could be transmitted from pets to people, even though the majority of such human infections are acquired from non-pet sources (i.e., *SalmonellaGiardia*, MRSA) were considered pet-associated zoonoses. Hepatitis was not considered pet associated, as Hepatitis A, B and C are non-zoonotic and Hepatitis E has only recently been thought to be transmitted from animals and the scientific community has yet to resolve if pets play any role in human infection [34,35].

We found considerable variation in the respondents’ ability to correctly classify different pathogens. As has been previously reported [27,28], the public appears to be well-informed of the potential for rabies transmission from pets (95% answered correctly), however, is less informed about less severe but more common pathogens, diseases and syndromes, such as ringworm, *Salmonella* and infectious diarrhea (53%, 33%, 24% answered correctly, respectively). Equally alarming, were the human or pet-specific diseases believed to be transmissible between species (i.e., HIV/AIDS, distemper, each 10%). As awareness of zoonotic disease risk is a prerequisite for effective prevention, the limited zoonotic disease knowledge of the public is a concern. Despite an increased awareness by researchers over the past several decades of the wide scope and magnitude of zoonotic diseases, the public’s knowledge appears to have changed little; our findings are consistent with those of a 1986 study [28] that reported similar proportions for the public’s correct classification of rabies, ringworm and infectious diarrhea as pet-associated zoonoses.

The low zoonotic disease awareness observed by respondents was perhaps not surprising as less than one-third of respondents reported having ever received information about pet-associated diseases or precautions to reduce the risk of these diseases. As previously noted [27,36], veterinarians and the Internet were most frequently reported as providing this information to pet and non-pet owners, respectively. Only 25% of pet owners recalled ever being asked by a physician or their staff if they owned pets. The limited involvement of physicians and public health was not surprising. Several studies have indicated that physicians often rely on veterinarians for advising the public about the potential for zoonotic disease and thus discuss this topic with their patients less frequently than veterinarians [37,38]. Although physicians believe educating patients about pet-associated health hazards is important, time constraints and competing health messages were often cited for not doing so [39]. Similarly, although veterinarians were an important source for zoonotic disease information in this study, there appear to have been many missed opportunities by the profession. Analogous to a previous study [27], only 27% of the individuals who had been to a veterinarian in the past year indicated they had ever received zoonotic disease information from a veterinarian.

Although statistically significant, the mean zoonotic disease knowledge score of pet owners was only marginally greater than non-pet owners (6.5, 6.2 respectively). The similar scores between the two groups implies that the additional resources available to pet owners (e.g., veterinarians, targeted education provided by physicians and public health) may not effectively provide a large amount of additional knowledge. Further efforts by these groups, such as readily available educational materials on this topic provided in waiting rooms and during office visits are important. A recent study reported that 43% of surveyed veterinarians did not have client educational materials on zoonotic diseases available in their practices [40]. Due to the ever-increasing use of the Internet for personal and animal health information [41], it is critical to ensure reliable resources are also available on-line.

From our results, it was evident that both the human and veterinary fields have room for improvement in patient/client education. Furthermore, increased communication between professions is needed to improve overall zoonotic disease knowledge and develop optimal approaches for reducing pet-associated pathogen transmission and injury in households. These conclusions are supported by previous studies that indicate that the majority of veterinarians and physicians do not regularly discuss zoonotic disease risks with clients, patients or each other [37,38,40,42]. The utility of such discussions was evident in our study, as respondents who recalled having received zoonotic disease information in the past had a higher mean zoonotic disease knowledge score compared to those who had not. While there was no assessment of the type, timing and quality of information that was provided, this finding suggests that general zoonotic disease counselling can have a positive impact.

Pet and non-pet owners were, for the most part, not concerned about pet-associated zoonoses and were comfortable with their level of knowledge and methods to reduce zoonotic disease risk. As individuals who are not concerned, and are comfortable with their knowledge-base may be unlikely to seek additional knowledge from available resources, active methods may be required to improve awareness of pet-associated zoonoses. Such methods may include brief waiting room surveys to assess disease knowledge and high-risk behaviours, followed by improved physician and veterinarian-directed review of pet-associated zoonoses with patients/clients. For pet owners, a significant positive association was observed between an individual’s zoonotic disease score and their level of comfort with their knowledge on this subject. Thus, for pet owners, inquiring about their level of comfort with this topic may be a reliable screening tool for identifying those in greatest need of additional education.

Pet ownership was common in households with individuals at higher risk of infections (e.g., < 5 yrs, ≥ 65 yrs, immunocompromised). Based on the limited studies that have previously surveyed this topic for the immunocompromised [15,20] and extremes of age [19,33,43,44], this finding was expected, and given the positive aspects of pet contact, this in itself was not particularly concerning. However, it was concerning that households with higher risk individuals did not differ from the remaining households regarding their perceived risk of pet-associated disease, zoonotic disease knowledge score, recalling being asked by a medical provider if they owned any pets, or recalling having received information regarding pet-associated disease risks and preventive measures. This suggests a troubling (but perhaps unsurprising) lack of recognition or knowledge of the potential for pet-associated zoonoses. A history of contact with pets in the home and animal contact outside the home should be part of every physician’s wellness evaluation, especially for individuals at higher risk of zoonotic disease. Primarily relying on veterinarians for providing pet-associated disease information is problematic for several reasons. As noted by our study, animal contact frequently occurred in both pet and non-pet owning households; many individuals with animal contact in the latter group will likely not consult a veterinarian. In addition, veterinarians are often unaware of their clients’ immune status [37,40] and arguably other attributes of higher risk households (such as extremes of age). Without this information, veterinarians are unable to adequately inform and council clients on their household disease risks. Veterinarians may also be reluctant to venture into aspects of human health.

A relatively low proportion (4%) of households reported having had a family member acquire a disease from a pet. The accuracy of this estimate is unclear, since it is prone to recall bias and various other factors that make it difficult to determine if an infection was truly acquired from a pet (e.g., multiple routes of transmission for many zoonotic pathogens, subclinical shedding of pathogens by pets). The potential risk of zoonotic infection and injury, however, were clearly noted in our study with a high proportion (27%) of households reporting one or more dog or cat-derived bites or scratches during the previous 12 months. This result is alarming, as such injuries can have serious health consequences. Previous studies vary widely in the reported incidence of animal bites, with most variation likely due to the population sampled and sampling methods used [23]. A U.S. study that used a telephone survey estimated a national annual incidence of self-reported dog bites of 1.8% (18 per 1000 population) [45]; by far the highest incidence reported to-date [23]. Our study found 1.6% of all household members sustained a self-reported dog bite during the previous 12 months. The high incidence of dog bites in our sample is unlikely to be due to differences in case definitions or sampling bias as we requested data only on bites that broke the skin; the proportions of pet ownership and presence of children in the households, two commonly reported risk factors for dog bites [23], were similar to previous reports and census data for the region. This finding warrants further evaluation by additional studies to confirm the elevated rate and determine potential risk factors. In the interim, educational resources addressing safe pet interaction and bite/scratch prevention strategies are strongly needed for this population.

We acquired our data as a convenience sample from the waiting rooms of general practice physician offices in Ontario, Canada. All Canadian residents receive medically necessary healthcare services at no charge [30], reducing the potential that variable access to physicians would result in a biased study population. Despite this, it is possible our source population or derived sample were not representative of the surrounding general population. Barriers in access to health care services that may disproportionately affect different groups, such as new immigrants [46], over-representation of groups typically in need of increased health care visits, such as the young, elderly, or immunocompromised, or use of a non-randomized sampling approach were potential concerns. However, based on census data, our sample appeared to be representative of the region, with the exception of a lower percentage of visible minorities and over representation of higher income households. The finding that 59% of households had one or more individuals at higher risk of infectious disease was reasonable as the proportions of our sample < 5 yrs (8%) and ≥ 65 yrs (8%) were consistent with census data for the region (6% and 12%, respectively); the proportion of households with immunocompromised members (32%) may be expected based on estimates for the United States [47] and the expected prevalence of immunocompromising conditions, such as diabetes [48], for the population in the Waterloo region.

Bias may have been introduced into our study. Possible sources of bias include differences between respondents and non-respondents (however the high response proportion of 75% makes this less likely) and the self-administered nature of the questionnaire where questions may have been misinterpreted (misclassification). In addition, respondents’ ability to recall information or activities typically deteriorates as time elapses, with better recall for more recent experiences. For this reason, it is possible the reported medical interactions (i.e., ever asked by a physician if they owned pets, ever received zoonotic disease information from a physician) are more reflective of the staff at the two surveyed clinics than other medical staff used by respondents previously. Due to the descriptive nature of the study, analysis relied primarily on univariable statistics, not accounting for possible correlation between the variables. Furthermore, pet ownership was associated with several demographics, as previously reported [27], that may have confounded the observed associations between pet ownership and attitudes, knowledge, or behaviours. Since this was a cross-sectional study, there are no data on the sequence of events relating to variables such as knowledge score.

We queried if respondents had pets in their household at the time of completing the survey and analyzed pet ownership as a dichotomous variable, not accounting for different pet species. Based on responses, it is likely that some of the non-pet owners previously had pets (i.e., 29% of non-pet owners who had previously received zoonotic disease information, had received this information from a veterinarian). It is possible this classification scheme may have biased our results, likely falsely increasing the zoonotic disease knowledge score attributed to non-pet owners and reducing apparent differences between pet and non-pet owners. Thus, differences between pet-owning households and those that have never owned pets may be more pronounced than we observed. In our analysis, we did not account for the ownership of different pet species. Different species have varying levels of disease risk for particular pathogens (e.g., high prevalence of *Salmonella* shedding by reptiles and amphibians), and owner demographics and other characteristics may vary by the species owned. Thus, it is possible that zoonotic disease knowledge, attitudes and practices may vary by species owned, and by classifying households simply as pet and non-pet owners systematic (bias) or nonsystematic error may have been introduced. However, since cats or dogs were owned by 93% of the pet owners and other species were infrequently reported, any such error was likely minimal.

## Conclusions

This study characterized household knowledge, attitudes and risks related to pet ownership and animal contact in Ontario, Canada. Despite frequent pet contact reported within and outside the house, awareness of the zoonotic risks from pets was limited and no greater in households with people at increased risk of infection. Educational efforts by human, veterinary and public health personnel were infrequently recalled. As awareness of disease risk is a prerequisite for effective disease prevention, further efforts by these key groups are needed, such as readily available educational materials provided in waiting rooms and accessible via the Internet, as well as active methods, such as discussions during office visits. Animal bite and scratch-based health risks are likely the greatest concern and materials should highlight these areas. Immediate educational efforts are especially needed for households with individuals at higher risk of infections. Given the time constraints on healthcare professionals, techniques such as waiting room surveys and newsletters may be helpful in initiating discussions with clients/patients. Both veterinarians and physicians are part of the family healthcare team and must work together to reach the common goal of reducing the public’s pet-associated disease risks. Finally, intervention studies, piloting various educational materials and methods for distributing these materials, are needed to determine the most effective ways to improve knowledge and reduce zoonotic disease risks.

## Competing interests

The authors declare they have no competing interests.

## Authors' contributions

All authors participated in the study concept, design and questionnaire development. JWS enrolled participating clinics and administered questionnaires to participating patients. JWS was responsible for data analysis, data interpretation and manuscript preparation. All authors provided input on data analysis, interpretation and final manuscript development. All authors have approved the final version of the manuscript.

## Pre-publication history

The pre-publication history for this paper can be accessed here:

http://www.biomedcentral.com/1471-2458/12/553/prepub

## Supplementary Material

Additional file 1 **Doc1 Questionnaire for households with pets.** Click here for file

Additional file 2 **Doc2 Questionnaire for households without pets.** Click here for file
