# Supplementary material for: Household knowledge, attitudes and practices related to pet contact and associated zoonoses in Ontario, Canada
Source: BMC Public Health. 2012 Jul 25;12:553. doi: 10.1186/1471-2458-12-553 (PMC3489606; doi:10.1186/1471-2458-12-553)
Supplement: Additional file 2 — Doc2 Questionnaire for households without pets. [file 1471-2458-12-553-S2.pdf]

Questionnaire for Households without Pets

(If you currently have pets in your household please notify study recruiter)

For the purpose of this survey, "**household pets**" is loosely defined to include pets that are indoor only, outdoor only, and those that spend time both indoor and outdoor.

Today's date (dd/mm/yyyy)_______________

Background

1. Which best describes where you live? (choose one)
City or urban [ ] Suburban [ ] Town or village [ ] Rural [ ]

2. Town/city in which you live:___________________

3. List the age and sex of each person that lives in your household and indicate if they live there full-time, part-time, or occasionally. List yourself first.

	Person's Age	Sex (Male/Female)	Lives in the house (check one)…		
			Full-time	Part-time (at least 50% of the time)	Occasionally (less than 50% of the time)
You→					

4. If there are children under 16 years of age in your household,

a. What is your relationship to them (parent, guardian, etc)? ____________________

b. How concerned are you that they could catch a disease from the pets of friends or family?

Very concerned [ ] Concerned [ ] Somewhat concerned [ ] Minimally concerned [ ] Not at all concerned [ ] Children do not have contact with pets [ ]

5. How concerned are you that you could catch a disease from the pets of friends or family?

Very concerned [ ] Concerned [ ] Somewhat concerned [ ] Minimally concerned [ ] Not at all concerned [ ] I do not have contact with pets [ ]

6. Please list the disease(s) which are of greatest concern to you____

7. To your knowledge, has anyone in your household ever caught a disease from a pet?
Yes [ ], please list the disease(s)____
No [ ]

Animal Contact

8. During the past 12 months, has anyone in your household been bitten or scratched by ANY dog or cat, where the skin was broken?
Yes [ ] No [ ] Don't know [ ]

a. If Yes, complete the table below

Age of person	Check all that apply for the past 12 months							
	Scratched by own dog	Scratched by another dog	Bitten by own dog	Bitten by another dog	Scratched by own cat	Scratched by another cat	Bitten by own cat	Bitten by another cat

9. Does anyone in your household regularly (at least weekly) have physical contact with animals in places outside of the home, such as at work, extracurricular activities, a friend's or school?
Yes [ ] (if ☑, indicate in table below the types of animals, where contact occurs, and age of household member involved. Two examples are provided.)
No [ ], (if ☑, proceed to question 10)

Type of animal	Where contact occurs	Age of household member involved
turtle	school	5 years
horse	riding lessons	35 years

10. Have any of your medical doctors or their staff ever asked if you owned any pets? Yes [ ] No [ ] Don't Remember [ ]

11. Have any of your medical doctors or their staff ever discussed the possible benefits of owning or keeping a pet? Yes [ ] No [ ] Don't Remember [ ]

12. Have you ever received information from any source about diseases that you can get from pets or precautions to take with pets to reduce the risk of disease?
Yes [ ] (if ☑, proceed to question 13)
No [ ] (if ☑, proceed to question 14)
Don't remember [ ] (if ☑, proceed to question 14)

13. Please indicate below which of the following sources provided you this information (check all that apply). For each that provided information, indicate if the information was useful or not useful.

	Provided information about diseases that can occur with pet contact	Was this information useful?		
		Useful	Not useful	Don't remember
Family physician				
Specialist physician				
Nursing staff				
Public health personnel				
Veterinarian				
Pet store				
Animal breeder				
Friends/relatives				
Internet				
Books				
Television/newspaper				
Other (list):				

a. Which of the above sources was most useful to you? (choose one)___________________________

14. Who do you believe should be responsible for providing information about diseases that can occur with pet contact? (check all that apply):

a. Family physician [ ]
b. Specialist physician [ ]
c. Nursing staff [ ]
d. Public health personnel [ ]
e. Veterinarian [ ]
f. Other (specify)___________________________________
g. None: I'm not concerned about anyone in my household getting diseases from pets [ ]

15. Please indicate how strongly you agree with the following statements (check one for each question):

	Strongly agree	Somewhat agree	Somewhat disagree	Strongly disagree	Unsure
a) I feel that pets are an important part of the family					
b) I feel that the benefits of owning a pet are greater than any health risks that occur with owning a pet					
c) I am comfortable with my level of understanding of possible diseases that can occur with pet contact					
d) I am comfortable with my level of understanding of ways to reduce diseases that can occur with pet contact					

16. Do you plan on acquiring a pet in the next year? Yes [ ] No [ ]

If Yes,

a. What type (cat, dog, etc)? List all:________________________________

b. From where do you plan to acquire it/them (check all that apply)

Pet store [ ]

Breeder [ ]

Shelter [ ]

Not yet sure [ ]

Other [ ] _______________________________

17. Please indicate which of the following diseases you think can be transmitted from pets to people?

Rabies	[ ]
Intestinal worms	[ ]
HIV/AIDS	[ ]
Distemper	[ ]
Salmonella	[ ]
Giardia	[ ]
Hepatitis	[ ]
Infectious diarrhea	[ ]
Ringworm	[ ]
Methicillin-resistant *Staphylococcus aureus* (MRSA)	[ ]
Measles	[ ]

Additional Background

18. Please indicate if anyone currently in your household has ever been diagnosed with any of the following conditions (check all that apply):

HIV/AIDS [ ]
Cancer of blood cells (such as leukemia) [ ]
Cancer of an organ (such as kidney cancer) [ ]
Other type of cancer [ ]
Cirrhosis of the liver [ ]
Diabetes [ ]
Other cause for immune system to not function properly [ ], please list_____________________________

Don't know [ ]
No one in my household has been diagnosed with any of these conditions [ ]

19. Before taxes and deductions, what is your total household income (from all household members and sources) during the past 12 months? (choose one)

Less than Can$20,000 [ ]
Between Can$20,000 and Can$39,999 [ ]
Between Can$40,000 and Can$79,999 [ ]
Between Can$80,000 and Can$120,000 [ ]
More than Can$120,000 [ ]

20. What is the highest level of education attained by anyone currently living in your household? (check one):

Elementary school [ ]
High school certificate, diploma, or equivalent [ ]
College, trade or other non-university certificate or diploma [ ]
University certificate, diploma or degree [ ]

21. Which of the following best describes you (choose only one):

a. White [ ]
b. Chinese [ ]
c. South Asian (e.g., East Indian, Pakistani, Sri Lankan, etc.) [ ]
d. Black [ ]
e. Filipino [ ]
f. Latin American [ ]
g. Southeast Asian (e.g., Vietnamese, Cambodian, Malaysian, Laotian, etc.) [ ]
h. Arab[ ]
i. West Asian (e.g., Iranian, Afghan, etc.) [ ]
j. Korean [ ]
k. Japanese [ ]
l. Other [ ], please specify_______________________________________

22. Were your children, you, or your parents born outside of Canada? Yes [ ] No [ ]

If <u>Yes</u>, indicate which individuals were born outside of Canada (check all that apply)

One or more children (if applicable) [ ]
You [ ]
One or more of your parents [ ]

This is the end of the survey. Thank you for agreeing to take part in this valuable study. Please feel free to use the space below for any additional comments regarding the survey or information you provided.
